# Cerebral venous thrombosis: direct thrombus imaging with sub-millimeter isotropic resolution dark-blood CMR

**DOI:** 10.1186/1532-429X-17-S1-P402

**Published:** 2015-02-03

**Authors:** Zhaoyang Fan, Qi Yang, Yibin Xie, Guoxi Xie, Xiaofeng Qu, Xiaoming Bi, Yutaka Natsuaki, Debiao Li

**Affiliations:** 1 Biomedical Imaging Research Institute, Cedars-Sinai Medical Center, Los Angeles, CA, USA; 2 Shenzhen Institutes of Advanced Technology, Shenzhen, China; 3 Siemens Healthcare, Los Angeles, CA, USA; 4 University of California, Los Angeles, CA, USA

## Background

Cerebral venous thrombosis (CVT) is a disorder potentially leading to devastating disability and even death if not timely diagnosed and treated. While TOF MR venography is most commonly used for diagnosis, the accuracy can be compromised by the flow voids caused by a slow or complex flow pattern and in-plane flow saturation. Several other MR techniques, relying on characteristic image contrast of CVT, may also under- or over-estimate the thrombus due to the signals from venous flow and other structures. High-spatial resolution dark-blood CMR could address the above issues, but, to our knowledge, has not been attempted. In this work, a fast dark-blood CMR technique was developed and validated in CVT patients.

## Methods

*Sequence* SPACE (variable-flip-angle 3D TSE) has increasingly been applied in vessel wall imaging at various arterial vascular beds due to its inherent dark-blood effect and fast imaging speed. However, suppressing slower venous blood flow remains a challenge for SPACE. To better detect CVT, particularly in the subacute stage, we combined T1-w SPACE with (a) a nonselective saturation pulse to exclude the T2-weighting that resides in the longitudinal magnetization at the end of the long echo train and (b) a DANTE preparation to suppress slow flow signals while introducing less T2-weighting. *Imaging* Using a 3T system and 32-ch coil, the sequence was first optimized for blood suppression on 5 healthy subjects (2F 3M). Six scans with different DANTE RF pulse train lengths (0, 50, 100, 150, 200, 250) were conducted with other DANTE parameters held fixed (FA 12^o^, RF gap 1ms, gradient strength 20mT/m). Imaging parameters for SPACE included: sagittal orientation, isotropic 0.78mm resolution, TR/TE 600/8.8ms, 37 echoes, GRAPPA factor 2, scan time 5min38sec. Contrast-to-noise ratio (CNR) analysis for the residual blood region vs. dark lumen and white matter vs. dark lumen were performed to determine the optimal DANTE pulse train length. The optimized sequence was prospectively tested on 4 patients (3F 1M) who were suspected of CVT.

## Results

**Figure 1:**
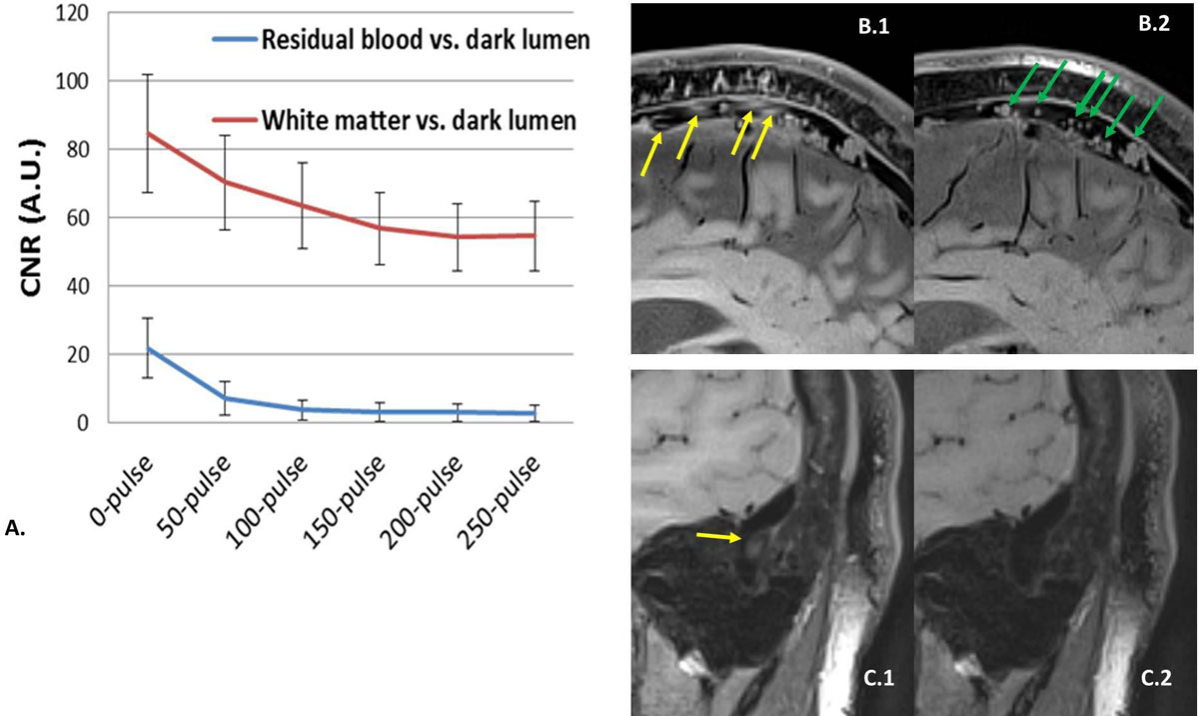
**Healthy volunteer study.** (A) The contrast-to-noise ratio (CNR) between the residual blood region and dark lumen decreases with the DANTE pulse train length. The CNR between the white matter and dark lumen has the similar trend. A pulse train length of 150 can adequately suppress venous blood signals while avoiding further signal loss in static tissues. (B) and (C) Representative images from two healthy subjects demonstrate that conventional SPACE imaging may have considerable flow artifacts that mimic thrombus denoted by yellow arrows in B.1 and C.1, whereas DANTE with 150 pulses can help dramatically reduce the artifacts (B.2 and C.2) and allow the visualization of normal sinus structures such as arachnoid granulations denoted by green arrows in B.2.

**Figure 2:**
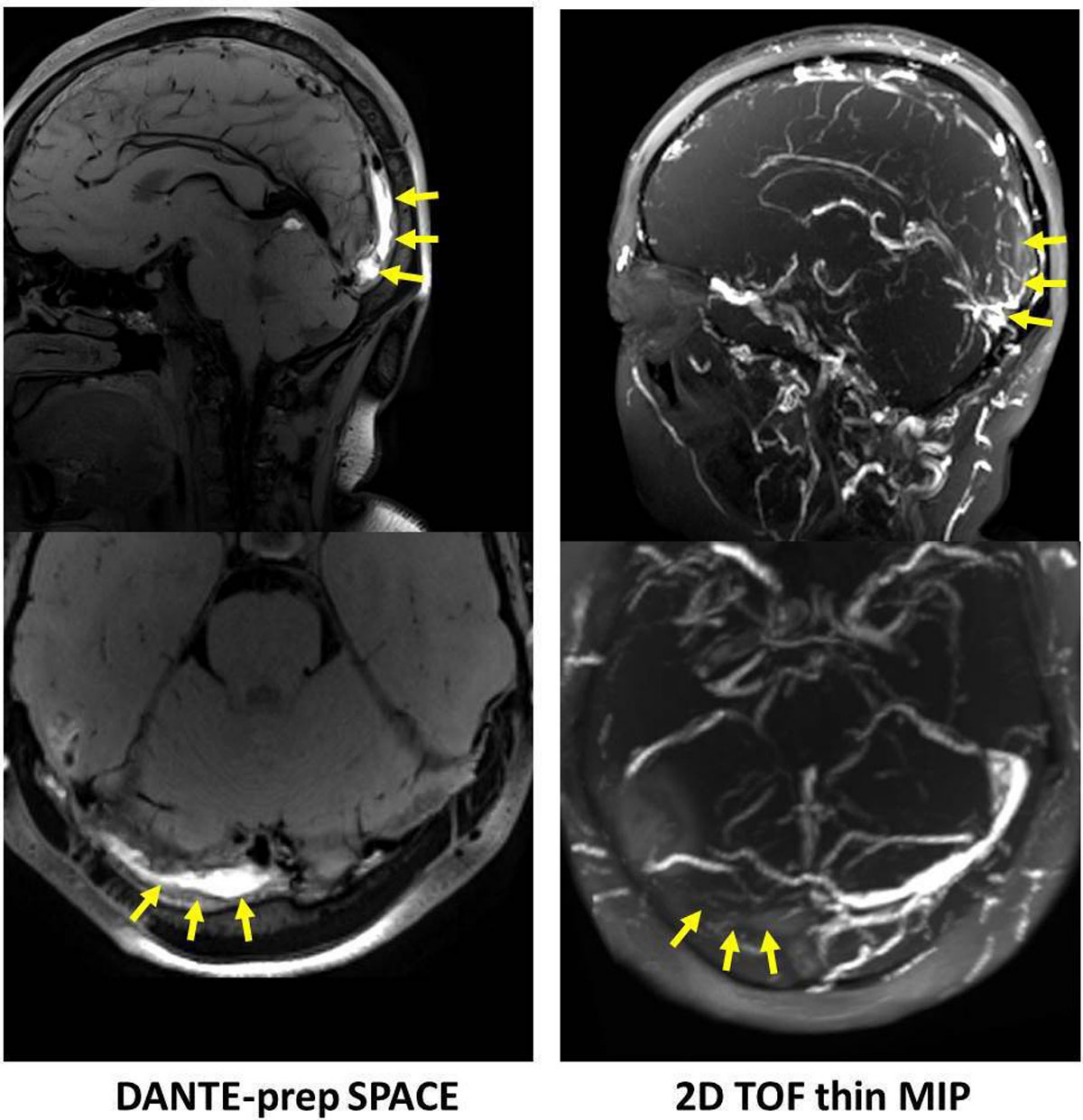
**Patient study.** Subacute CVT at the superior sagittal sinus and right transverse sinus is clearly depicted by DANTE-prepared T1-weighted SPACE. Note that hyper-intense signals on SPACE match flow voids on TOF as denoted by yellow arrows. The size of CVT is better appreciated on SPACE because of the positive contrast.

Without DANTE, all healthy subjects exhibited residual venous flow signals in at least one venous sinus (Fig. 1B.1&C.1). CNR analysis indicated that DANTE with 150 pulses appeared to be a suitable preparation to yield sufficiently clean sinus lumens while avoiding further signal loss in static tissues (Fig. 1A). The 0.78-mm isotropic resolution dark-blood images allowed identification of fine normal structures (e.g. arachnoid granulations in Fig. 1B.2) in sinuses. Subacute CVT was clearly depicted as hyper-intense substances in all patients (Fig. 2).

## Conclusions

This work, for the first time, investigated the feasibility of dark-blood CMR for CVT imaging. The T1-w DANTE-prepared SPACE technique eliminates the flow artifact in the sinus and permits direct visualization of intrasinus thrombus and surrounding structures. A large clinical study is underway to validate the clinical value of the technique for different CVT stages.

## Funding

N/A.

